# BDNF and TrKB expression levels in patients with endometriosis and their associations with dysmenorrhoea

**DOI:** 10.1186/s13048-022-00963-9

**Published:** 2022-03-17

**Authors:** Sha Wang, Hua Duan, Bohan Li, Wei Hong, Xiao Li, Yiyi Wang, Zheng Chen Guo

**Affiliations:** Department of Minimally Invasive Gynecologic Center, Beijing Obstetrics and Gynecology Hospital, Capital Medical University, Beijing, 100006 China

**Keywords:** BDNF, TrKB, Endometriosis, endometrium, Dysmenorrhoea

## Abstract

**Background:**

Brain-derived neurotrophic factor (BDNF) is a known regulator of the development and maintenance of chronic pain in various chronic disorders. Together with its high-affinity tyrosine kinase type B (TrKB) receptor, BDNF is extensively expressed in the mammalian female reproductive system. However, BDNF and TrKB expression in different stages of endometriosis and the relationship between the expression of each in ectopic lesions and endometriosis pain remain unclear.

**Methods:**

Sixty-two women who underwent laparoscopic surgery were enrolled in this study: forty-six diagnosed with ovarian endometrioma (study group) and sixteen diagnosed with ovarian benign tumours (control group). Samples from eutopic endometrium and ovarian endometriotic lesions were obtained at laparoscopic surgery. BDNF and TrKB messenger RNA (mRNA) and proteins levels in the eutopic and ectopic endometrium of both groups were measured by real-time PCR and immunohistochemical staining, respectively. Before the surgery the visual analogue scale (VAS) was used to measure dysmenorrhoea.

**Results:**

BDNF and TrKB expression levels were higher in ovarian endometriotic lesions than in eutopic endometrium and normal endometrium (*P* < 0.05), and there was no cyclical change. Furthermore, their expression levels were higher in eutopic endometrium than in normal endometrium (*P* < 0.05), and BDNF and TrKB levels were higher in stage IV ovarian endometriotic lesions than in stage II and III lesions (*P* < 0.05), with their expression being non-significantly higher in stage III than in stage II (*P* > 0.05). Additionally, correlation coefficients for the association analysis between the mRNA expression of BDNF or TrKB in eutopic endometrium and the dysmenorrhoea VAS score were *r* = 0.52 and *r* = 0.56 for BDNF and TrKB, respectively (*P* < 0.05). The correlation coefficients for the associations between BDNF and TrKB in both the eutopic and ectopic endometrium were *r* = 0.82 and *r* = 0.66, respectively (*P* < 0.05).

**Conclusions:**

BDNF and TrKB are closely related to dysmenorrhoea caused by endometriosis and may be important in the pathobiology or pathophysiology of endometriosis.

## Introduction

Endometriosis is one of the most common chronic gynaecological conditions and is characterized by the presence of endometrial-like tissue that undergoes proliferation, bleeding and regeneration outside the uterine cavity. This disease has become one of the most important causes of infertility and pelvic pain, affecting 6–10% of women of reproductive age [1]. Although there are several, though not fully confirmed theories on the pathogenesis of endometriosis, the exact aetiology of endometriosis remains unclear. A recent study showed that endometriotic lesions are caused by repeated tissue injury and repair (TIAR) [2]. TIAR is considered an evolutionarily conserved system that is independent of steroidal precursors usually produced in endocrine glands such as the adrenal gland and gonads. Increased inflammatory mediators such as interleukin (IL) 1β, IL6, tumour necrosis factor α, and prostaglandins are thought to sensitize sensory neurons by stimulating the sensory nerve fibres within the ectopic lesions, thus triggering the pain signal cascade in women with endometriosis. Endometriosis pain may be considered a kind of inflammatory and neuropathic pain [3].

Brain-derived neurotrophic factor (BDNF) is a member of the neurotrophin family of secreted growth factors that are classically involved in the development, growth and function of both central and peripheral neurons. BDNF plays an important role in nociceptive and neuropathic pain [4]. BDNF is a regulator that is involved in the formation and maintenance of chronic pain in various chronic disorders, including osteoarthritis [5], rheumatoid arthritis [6], fibromyalgia [6, 7], and facet joint distraction [8]. BDNF, together with its high-affinity tyrosine kinase type B (TrKB) receptor, is extensively expressed in the mammalian female reproductive system [9]. Increased serum BDNF concentrations have been observed in patients with endometriosis with central sensitivity syndrome. Additionally, the neurotrophic protein family has been demonstrated to be important in the pathophysiology of endometriosis by several independent investigators. One study found that initially, endometriosis patients had higher levels of BDNF in circulation than the control group of patients without endometriosis [10], and this finding was subsequently replicated by another group [11]. BDNF circulating levels were later associated with pelvic pain scores in another group of women with endometriosis compared with the relevant control group [12]. Ding and colleagues [13] went on to confirm the role of BDNF in endometriosis and pelvic pain in their observation of higher BDNF levels in the peritoneal fluid of women with endometriosis and pelvic pain compared to the control group. Furthermore, they confirmed differences in BDNF expression among lesion types.

In the present study, we aimed to measure the expression levels of BDNF and TrKB in the eutopic endometrium and ovarian endometriotic lesions of patients with endometriosis and to analyse the differences in expression among patients with and without endometriosis. Subsequently, we evaluated the expression patterns of BDNF and TrKB at different stages of endometriosis and examined whether there are correlations between BDNF or TrKB and endometriosis severity or dysmenorrhoea, with the goal of providing a theoretical basis for the aetiological mechanism of endometriosis and the treatment of endometriosis-related pain.

## Methods

### Patients and specimens

This study included patients diagnosed with endometriomas and ovarian benign tumours who were referred to our centre from May 2017 to July 2018. The study was approved by the local research and ethics committee of Beijing Obstetrics and Gynaecology Hospital (No. 2016-KY-01). Written informed consent was obtained from each patient before sampling. The study group included forty-six patients who underwent laparoscopic surgery for ovarian endometrioma and received a histopathological diagnosis at Beijing Obstetrics and Gynaecology Hospital. Endometriosis was surgically and histologically diagnosed as stage I, II, III, or IV according to the revised American Fertility Society (r-AFS) classification scheme [14]. The demographic and clinical characteristics of the patients were as follows: 6 had stage II endometriosis, 3 in the proliferative phase and 3 in the secretory phase, and the mean age was 33.70 ± 5.80 years; 19 had stage III endometriosis, 9 in the proliferative phase and 10 in the secretory phase, with a mean age of 33.20 ± 6.20 years; and 21 had stage IV endometriosis, 9 in the proliferative phase and 12 in the secretory phase, with a mean age of 32.50 ± 6.40 years. The differences in age among these patient groups were not statistically significant (*P* > 0.05). The exclusion criteria were as follows: patients with no history of a sexual life, with hormonal medicine use within 3 months before surgery, or with adenomyosis, polycystic ovary syndrome (PCOS), pelvic inflammatory disease, endometrial lesions, genital dysplasia, malignant tumours of any organ, pregnancy or neurological diseases. In patients undergoing laparoscopic surgery, the cyst was completely detached under the laparoscope, the contents were aspirated, and a small amount of the cyst wall was then cut under strict aseptic conditions; this sample was used to isolate and culture primary ectopic endometrial cells, and parts of the sample were paraffin-embedded and cryopreserved.

Sixteen patients of reproductive age with laparoscopic surgery for benign ovarian tumours and who were diagnosed by histopathology or who had no endometriosis were recruited as the control group. Eight were in the proliferative phase, the other 8 were in the secretary phase, and the mean age was 33.40 ± 7.00 years. The difference in age between this group and the endometriosis group was not statistically significant (*P* > 0.05). The exclusion criteria were the same as those for the endometriosis group. The basic characteristics of the two groups of patients are shown in Table 1. Eutopic endometrial and ovarian endometriotic lesions were taken from the endometriosis group, and normal endometrium of approximately 1 cm × 1 cm × 0.5 cm in size was obtained from each subject in the control group.

**Table 1:** Overview of the demographics and other characteristics of the recruited patients

**Parameter**	**Endometriosis** ***N* = 46**	**Control** ***N* = 16**	***P***
**Age, mean (SD)**	32.97 (7.23)	33.40 (7)	0.74
**Gravidity, median (range)**	2 (0, 5)	2 (1, 5)	0.76
**Parity, median (range)**	1 (0, 5)	1 (0, 2)	0.59
**Haemoglobin, mean (SD)**	102.8 (27)	120.9 (15.2)	0.01
**CA125, mean (SD)**	55.8 (5.2)	13.32 (6.24)	0.995
**PBAS, mean (SD)**	158.7 (67.6)	112.9 (25.9)	0.12
**Menstrual cycle (Proliferative phase) n (%)**	21 (45.7%)	8 (50%)	0.98
**Leiomyomas n (%)**	**12 (26.1%)**	**5 (31.2%)**	**0.76**
**Endometrial polyps n (%)**	**19 (41.3%)**	**8 (50%)**	**0.796**

*Abbreviations: PBAS* Pictorial Blood Loss Assessment Chart Score

Differences among groups were analysed by Student’s t-test and the Mann–Whitney U test

### Pain (dysmenorrhoea) evaluation

Was used before surgery to evaluate. The presurgical severity of dysmenorrhoea in patients with endometriosis was evaluated by means of the VAS (0–10 score, 0 = no pain and 10 = maximum pain). The VAS is considered the ‘gold standard’ for pain measurement and can be used for multiple types of pain, including dysmenorrhoea, dyspareunia, dyschezia and chronic pelvic pain [15]. VAS scores were grouped into three levels: mild (ranging from 0 to 3), moderate (ranging from 4 to 6), and severe (ranging from 7 to 10).

### Immunohistochemistry and quantification by H-score

All samples were fixed with 10 mM PBS-buffered 10% formalin, embedded in paraffin, and cut into sections of 4-μm thickness. For heat-induced epitope retrieval, deparaffinized sections in 0.01 mmol/L citrate buffer were heated for 20 min at 95 °C using a microwave oven. Immunohistochemical staining was carried out according to the avidin–biotin immunoperoxidase method using the UltraSensitiveTM S-P kit (Maixin Corporation, China). Endogenous peroxidase activity was blocked for 10 min with 50 μl peroxidase blocker from the UltraSensitive™ S-P kit, followed by washing with PBS 3 times for 2 min each time. The sections were incubated at 4 °C overnight with the following primary antibodies: anti-BDNF (1:50 dilution, ab108319; Abcam, Cambridge, MA, USA) and anti-TrKB (1:100 dilution, ab108319; Abcam, Cambridge, MA, USA). The sections were then rinsed and incubated with biotinylated secondary antibodies for 10 min. After washing with PBS, the sections were further incubated with horseradish peroxidase-conjugated streptavidin for 5 min and finally treated with diaminobenzidine in 0.01% H_2_O_2_ for 5 min. The slides were counterstained with Mayer’s haematoxylin, and the stained sections were observed under a microscope (Axio Imager 2, Zeiss, Oberkochen, Germany). The immunoreactive intensity of the BDNF- and TrKB-stained cells was quantified by a modified method of histogram scoring (H-score) as described previously [16]. The staining intensity was graded as follows: 0, no staining; 1, weak; 2, moderate; or 3, strong. A total percentage score (% of cells with staining intensity ≥ 1, namely, the sum of the percentage of cells with intensities of 1, 2, and 3) was used to semiquantitatively evaluate the tissue expression levels of BDNF and TrKB. The H-score was calculated using the following formula: H-score = [(% at 0) × 0] + [(% at 1 +) × 1] + [(% at 2 +) × 2] + [(% at 3 +) × 3].

### Statistical analysis

Statistical analysis was performed using Statistical Product and Service Solutions (SPSS) 22.0. Quantified data are expressed as the mean ± standard deviation. Comparisons between groups were performed with independent-sample t tests. Analysis of variance (ANOVA) was used to compare the means of more than two groups. The correlations among BDNF and TrKB expression and the severity of dysmenorrhoea were assessed using Spearman’s correlation. *P* values of < 0.05 were considered significant.

## Results

### BDNF and TrKB protein expression levels in the endometriosis and control groups

**Fig. 1:**
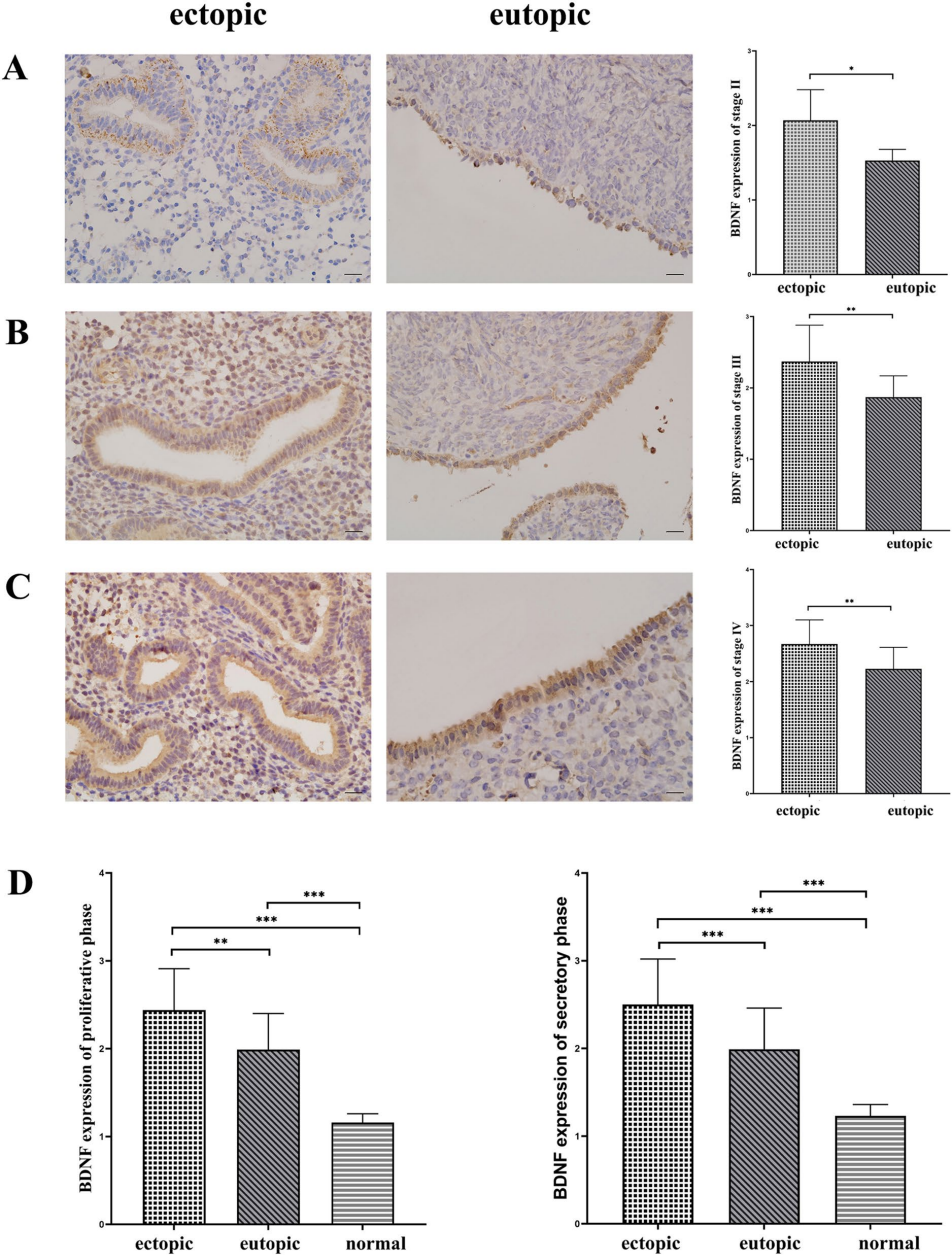
Immunohistochemical staining of BDNF. (immunohistochemical staining, × 400, scale 20 μm). The expression of BDNF in eutopic and ovarian ectopic endometria of the endometriosis group was positive, which was indicated by yellow, brown and tan particulates, mainly discovered in the cytoplasm of epithelial cells or glandular epithelial cells. **A** Ectopic and eutopic endometrial staining and H-score of BDNF in stage II. **B** Ectopic and eutopic endometrial staining and H-SCORE values of BDNF in stage III. **C** Ectopic and eutopic endometrial staining and H-SCORE values of BDNF in stage IV. **D** H-SCORE values of BDNF expression of both proliferative (left) and secretory (right) phases for ectopic and eutopic endometrium in the endometriosis group and normal endometrium in the control group. *, *P* < 0.05; ∗ ∗ , *P* < 0.01; ∗ ∗ ∗ , *P* < 0.001

**Fig. 2:**
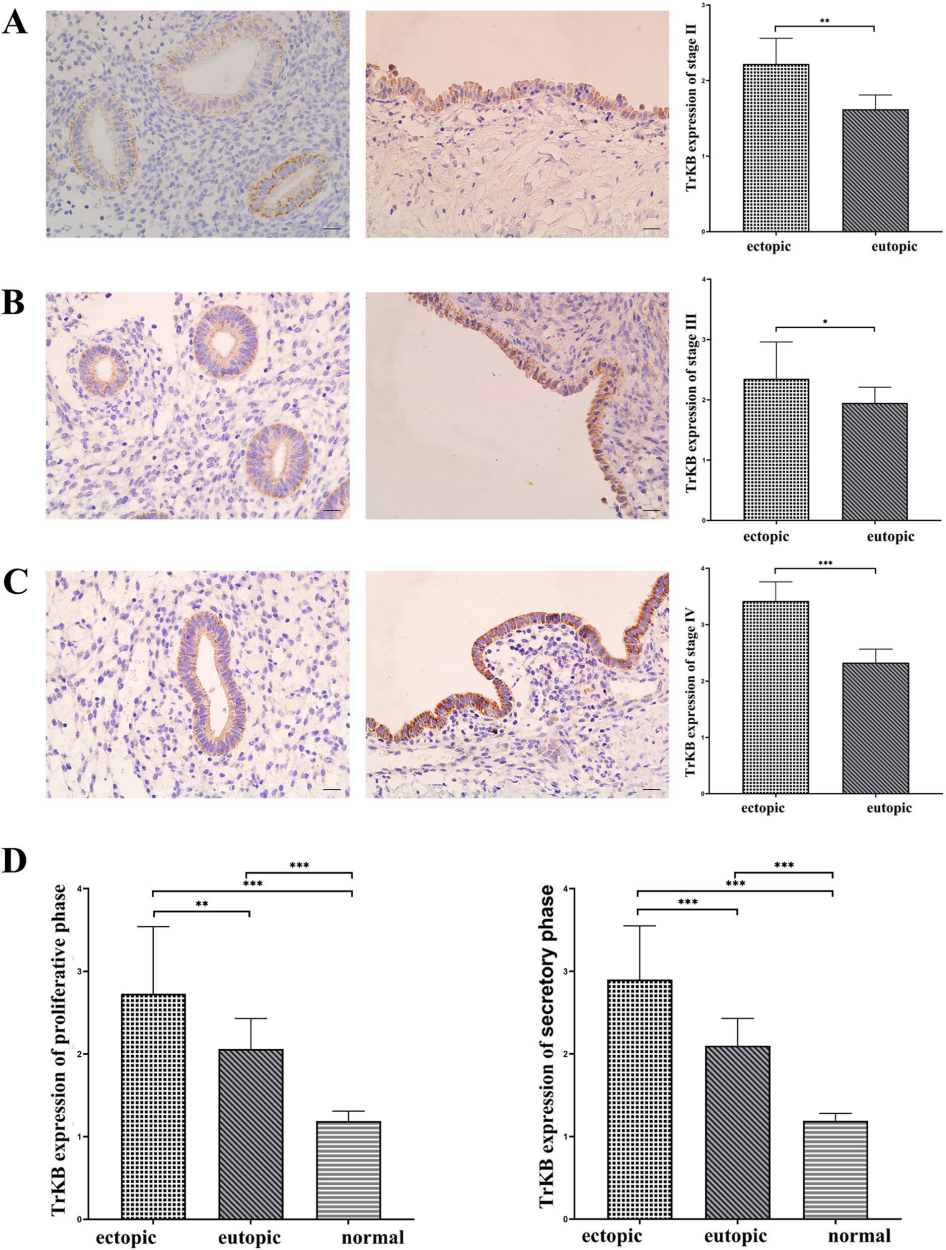
Immunohistochemical staining of TrKB. (immunohistochemical staining, × 400, scale 20 μm). The expression of TrKB in eutopic and ovarian ectopic endometria of the endometriosis group was positive, which was indicated by yellow, brown and tan particulates, mainly discovered in the cytoplasm of epithelial cells or glandular epithelial cells. **A** Ectopic and eutopic endometrial staining and H-SCORE values of TrKB in stage II. **B** Ectopic and eutopic endometrial staining and H-SCORE values of TrKB in stage III. **C** Ectopic and eutopic endometrial staining and H-SCORE values of TrKB in stage IV. **D** H-SCORE values of TrKB expression of both proliferative (left) and secretory (right) phases for ectopic and eutopic endometrium in the endometriosis group and normal endometrium in the control group. *, *P* < 0.05; ∗ ∗ , *P* < 0.01; ∗ ∗ ∗ , *P* < 0.001

BDNF and TrKB were stained in normal endometrium, eutopic endometrium, and ectopic endometrium. In both proliferative and secretory phases, BDNF had the highest protein expression level in ectopic endometrial tissue, followed by eutopic and normal endometrium (*P* < 0.05). The same was true for the expression of TrKB (*P* < 0.05). As shown in Fig. 1D and 2D. However, when comparing the expression of BDNF and TrKB between the proliferative and secretory phases, there was no significant difference in either ectopic or normal endometrium (*P* > 0.05).

### Expression of BDNF and TrKB in different stages of endometriosis

Afterwards, the expression of BDNF in eutopic and ectopic endometria at each stage was analysed. In patients with stage II endometriosis, the expression of BDNF in the ectopic endometrium was significantly higher than that in the eutopic endometrium (*P* < 0.05). In patients with endometriosis stages III and IV, the expression of BDNF in the ectopic endometrium was also higher than that in the eutopic endometrium (*P* < 0.05). As shown in Fig. 2.

Furthermore, the analysis of the expression of BDNF in each stage of the ectopic endometrium showed that the expression of BDNF in stage IV was significantly higher than that in stage III and II. The expression in phase III was also significantly higher than that in phase II (*P* < 0.05). However, the expression of BDNF in phase III was not significantly different from that in phase II (*P* > 0.05). Meanwhile, in the analysis of BDNF expression in the eutopic endometrium in each stage, it was found that as the stage increased, its expression gradually increased (*P* < 0.05).

Similarly, in the analysis of TrKB expression in each stage, we found the same pattern. In patients with stage II, stage III and stage IV endometriosis, TrKB expression was found to be higher in ectopic endometrium than in eutopic endometrium (*P* < 0.05), as shown in Fig. 2. In eutopic and ectopic endometria of patients with endometriosis, TrKB expression increased with increasing stage. There were significant differences in TrKB expression in each phase of the eutopic endometrium (*P* < 0.05). In the analysis of TrKB expression in ectopic endometrium, there were significant differences between each stage (*P* < 0.05) but not between stage III and stage II (*P* > 0.05).

### The correlation between mRNA expression of BDNF and TrKB in the endometriosis group and dysmenorrhoea VAS score

**Fig. 3:**
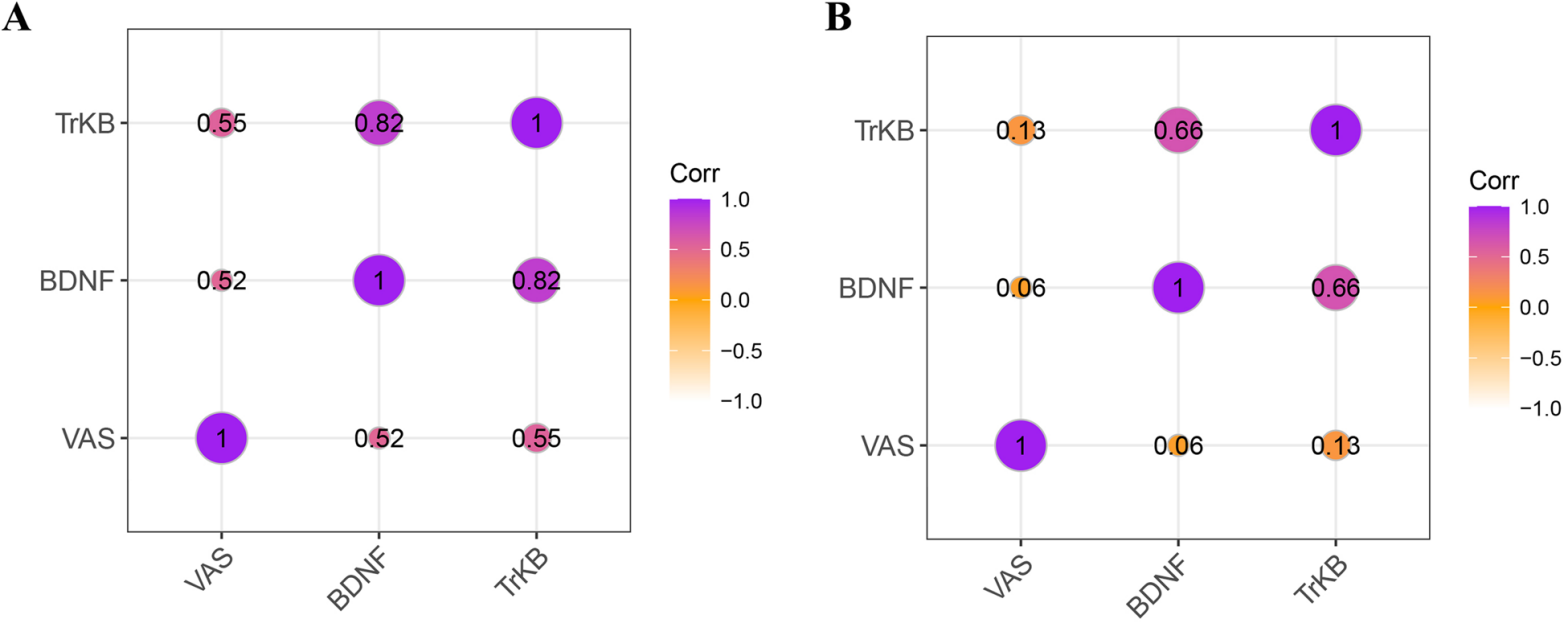
Correlation between mRNA expression of BDNF, TrKB in endometriosis group and dysmenorrhea VAS score. **A** mRNA expression of BDNF, TrKB in eutopic endometrium and the correlation among BDNF, TrKB and dysmenorrhea. **B** mRNA expression of BDNF, TrKB in ectopic endometrium and the correlation among BDNF, TrKB and dysmenorrhea

To analyse the correlations among BDNF, TrKB and dysmenorrhoea, the mRNA expression levels of BDNF and TrKB in the endometriosis group were assessed, as shown in the Fig. 3. The Spearman rank correlation coefficient for the association between BDNF mRNA expression in the eutopic endometrium of the endometriosis group and the dysmenorrhoea VAS score was *r* = 0.52, demonstrating that there was a moderate positive association between BDNF expression in the eutopic endometrium and dysmenorrhoea VAS score *(P* < 0.05); however, there was no association between BDNF expression in ovarian endometriotic lesions and the dysmenorrhoea VAS score (*P* > 0.05).

The Spearman rank correlation coefficient of the association between TrKB expression in the eutopic endometrium of the endometriosis group and the dysmenorrhoea VAS score was *r* = 0.56, meaning there was a moderate positive association between TrKB expression in the eutopic endometrium and the dysmenorrhoea VAS score (*P* < 0.05); however, there was no association between TrKB expression in ovarian endometriotic lesions and the dysmenorrhoea VAS score (*P* > 0.05).

Pearson analysis coefficients for the associations between BDNF and TrKB in both eutopic and ectopic endometrium were *r* = 0.82 and *r* = 0.66, respectively (*P* < 0.05).

## Discussion

Accumulative evidence suggests that immune cells, adhesion molecules, extracellular matrix metalloproteinase and proinflammatory cytokines activate and alter the peritoneal microenvironment, creating the conditions for differentiation, adhesion, proliferation and the survival of ectopic endometrial cells. Latanà et al. [17, 18]. have described the developmental process of endometriosis through different perspectives. Briefly, the retrograde shedding and implantation of endometrial stem cells and the epigenetic regulatory changes that occur as a result of receiving relevant environmental influences are the basis for their pathogenesis. In contrast, alterations in the peripheral immune microenvironment, including macrophages and NKT cells, are important advancing factors in disease development [19].

There are many theories on the pathogenesis of endometriosis, but the menstrual blood reflux theory proposed by Sampson still occupies the leading position [20]. A study found that BDNF was detected in menstrual blood and endometrium in reproductive-aged females, with quantitative analysis showing that the concentration of BDNF in menstrual blood is higher than that in plasma [21], indicating that BDNF may play a role in female reproductive function. TrKB expression is increased in the eutopic endometrium of patients with endometriosis, and TrKB is considered to be one of the most potent growth factors for inhibiting anoikis [22]. Our study found that the endometriotic endometrium and non-endometriotic endometrium were significantly different in terms of BDNF and TrKB expression, which is consistent with the literature [23, 24]. Moreover, this study showed that the expression levels of BDNF and TrKB in ovarian endometriotic lesions were higher than those in eutopic endometrium, which is similar to the finding by Borghese et al., who showed that BDNF expression in ovarian endometriotic lesions was higher than that in eutopic endometrium. This suggests that BDNF and TrKB may be important in the pathobiology or pathophysiology of endometriosis.

Previous studies have found that in mice, oestrogen exposure after ovariectomy significantly upregulated BDNF, but the hormonal fluctuations of the murine oestrous cycle did not [25]; evaluations of the plasma BDNF concentrations of endometriosis patients by ELISA showed that BDNF expression was not affected by the menstrual period [26]; other studies suggested that TrKB expression in eutopic endometrium has no correlation with the menstrual period [27]. Our results showed that there was no significant difference in BDNF or TrKB expression between the proliferative and secretary phases of the menstrual cycle in either ovarian endometriotic lesions or eutopic endometrium in endometriosis patients.

The study also found that there was a significant difference in the degree of adhesion and infiltration depth between different stages of endometriosis, and the degree of adhesion and infiltration depth also increased with increasing stage. The results of our study showed that BDNF and TrKB expression in ovarian ectopic lesions and eutopic endometrium increased with increasing r-AFS stage, suggesting that BDNF and TrKB may be correlated with the severity of endometriotic lesions. Additionally, BDNF and TrKB expression increased with increasing stages and resulted in the neogenesis of ectopic lesions and aggravated the progression of the original lesions continuously by inhibiting anoikis and promoting cell invasion, proliferation and angiogenesis. Furthermore, the study demonstrated that ectopic lesions can synthesize BDNF continuously [28, 29], thus forming a vicious cycle and further leading to aggravation of the disease.

Consistent with previous studies, we found that BDNF and TrKB expression in ovarian endometriotic lesions was not correlated with the dysmenorrhoea VAS score [30]. We also found that there was a moderate positive correlation between BDNF or TrKB expression in the eutopic endometrium and the dysmenorrhoea VAS score. Therefore, we can speculate that the characteristics of the eutopic endometrium play a key role in dysmenorrhoea in endometriosis and that the effect of molecular biological changes in the eutopic endometrium on the genesis of dysmenorrhoea in endometriosis may be larger than that of the ovarian endometriotic lesions, suggesting that BDNF and TrKB may be correlated with dysmenorrhoea in endometriosis. The significant increase in BDNF in endometriosis and the abnormal distribution of different types of nerve fibres may be the causes of endometriosis-associated pain. Together, BDNF, vascular growth factor and immune inflammation factor are involved in the peripheral nerve sensitization caused by chronic activation of nerve endings.

This study further validated the effect of the BDNF/TrKB signalling pathway on the process of endometriosis and analysed changes in the expression of these factors, together with associated changes in the degree of dysmenorrhoea, in different parts of the endometrium at different stages.

**Fig. 4:**
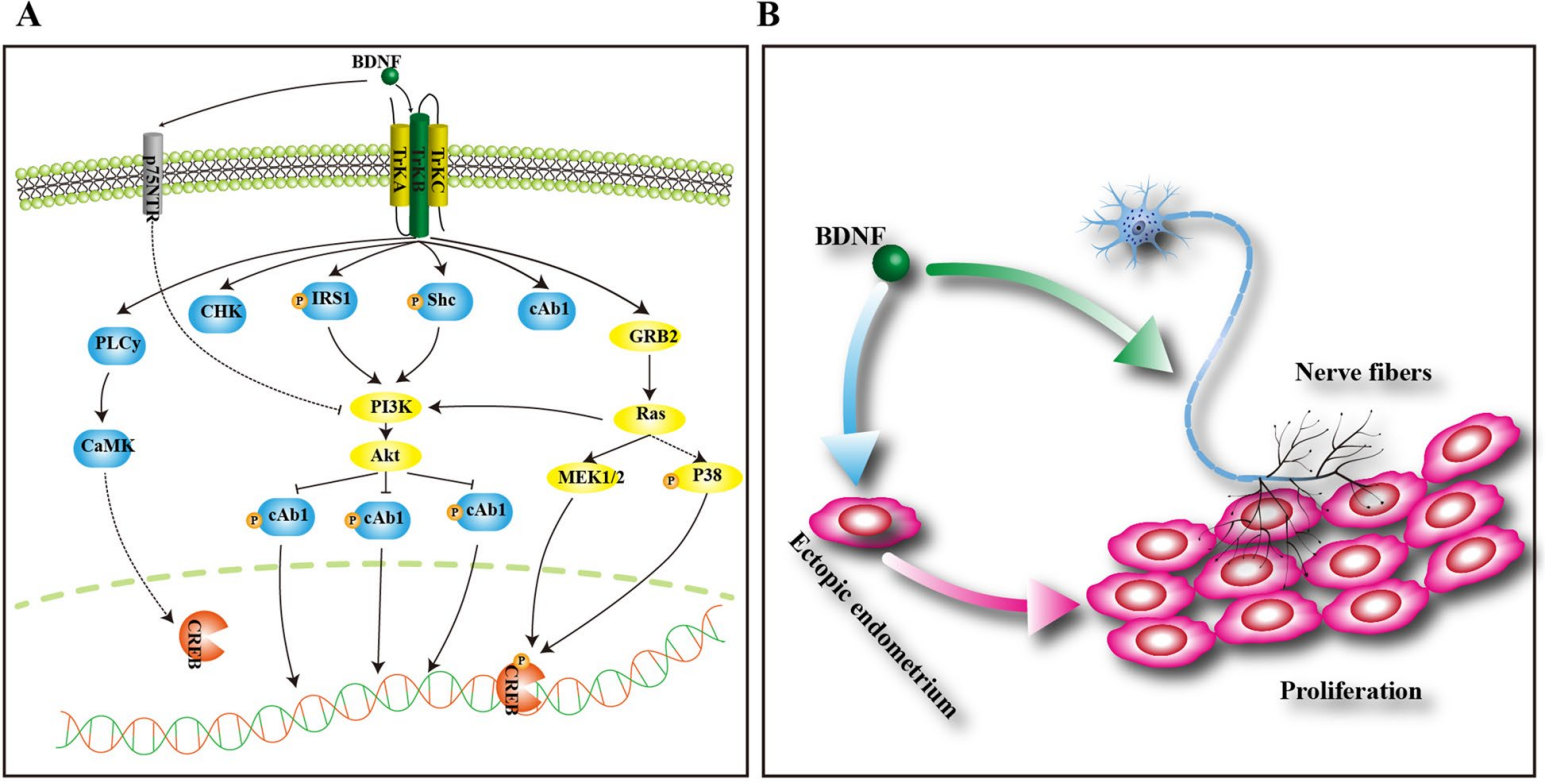
Diagram of the molecular mechanisms of BDNF. **A** BDNF functions via engagement of TrKB or p75. Neurotrophin/Trk signalling is regulated via connections among a variety of intracellular signalling cascades, including the MAPK pathway, PI-3 kinase pathway, and PLC pathway, transmitting positive signals that enhance survival and growth. On the other hand, p75 transmits both positive and negative signals. **B** BDNF may be synthesized in ectopic endometrium, which can promote the growth of nerve fibres and endometrial cells

Pelvic pain is the most typical clinical symptom in endometriosis, but the exact mechanism remains unclear. At present, the three main mechanisms for pain include the close connection between lesions and surrounding nerves, the specific inflammatory environment and the effect of the central nervous system on pain [31]. As an important member of the NT family, BDNF has a strong effect on the growth, differentiation and survival of nerve fibres [32] and promotes visceral pain and high sensitivity [33]. One study found that there were no nerve cells in endometriotic lesions; however, BDNF and NGF could promote the generation of nerve tissue in endometriotic lesions [34], and the expression levels of BDNF and NGF were correlated with the degree of pain in endometriosis patients [35]. However, another study showed that the expression levels of BDNF and NGF were not significantly correlated with the degree of pain [36]. The manifestations of endometriosis-associated pain for different types and locations vary, and the mechanisms are different between peritoneal endometriosis and deep infiltrating endometriosis (DIE). The former may be related to the stimulation of cytokines and growth factors generated by lesions, while the latter is related to the stimulation of various factors, as well as deep lesions, as reported in a previous study [37] (Fig. 4).

A recent study has shown that women may have disease for many years before diagnosis, be seen by multiple health care providers before obtaining a diagnosis and undergo multiple laparoscopies over the course of their disease [38]. In our study, only three patients had previous surgical therapy for endometriosis. Most of the patients were initially diagnosed with endometriosis in our hospital and underwent surgery because they had no significant symptomatic improvement after pharmacological treatment. It is also noteworthy that although there were no statistically significant differences in baseline characteristics between patients with endometriosis and control patients in this study, haemoglobin levels were significantly different. This may be closely associated with their longer menstrual period as well as their increased menstrual volume. Additionally, whether this difference affects BDNF expression deserves further investigation in subsequent studies.

Additionally, there are some shortcomings in this study. The sample size is small and too small to reach conclusions between groups with stratification by cycle stage. Furthermore, the sample size is not sufficient to achieve firm conclusions, as this can only be considered a pilot study. Moreover, limited by the case data, we have information only about the patient’s dysmenorrhoea. We will address these limitations in a follow-up study. However, our previous study was exploratory, with a small sample size, a lack of r-AFS stage I patients when collecting samples and a small number of r-AFS stage II patients due to the preliminary nature of this exploratory research. In the future, we will expand the sample size and systematically explore the mechanism of the Neurotrophin/Trk signalling pathway and its effect on endometrial and nerve fibre cells.

## Conclusion

The present study demonstrated that BDNF and TrKB may be associated with endometriosis severity. The expression of BDNF and TrKB in the eutopic endometrium of endometriosis increased with the increase in dysmenorrhoea VAS, but there were no correlations between BDNF or TrKB and the dysmenorrhoea VAS score for patients with endometriotic lesions. This finding may indicate that BDNF and TrKB may be important in the pathobiology or pathophysiology of endometriosis. However, this study is limited to the assessment of a single time point in the course of a woman’s endometriosis journey, and the important role of BDNF and TrKB in promoting the mechanism of endometriosis remains to be further studied.

## Data Availability

All analysis results are displayed in the results. For specific experimental data, please contact the corresponding author.

## Abbreviations

BDNF: Brain-derived neurotrophic factor
TrKB: Tyrosine receptor kinase B
mRNA: Message RNA
VAS: Visual analogue scale
NT: Neurotrophin
r-AFS: Revised American Fertility Society
PCOS: Polycystic ovary syndrome
H-score: Histogram scoring
ANOVA: Analysis of variance
